# Health system and policy response to climate change in the West Bank, Palestine: Current situation and priority actions

**DOI:** 10.1371/journal.pgph.0005617

**Published:** 2025-12-08

**Authors:** Maysaa Nemer, Mais Araj, Hanin Basha, Yasmeen Wahdan, Niveen Abu-Rmeileh

**Affiliations:** 1 Institute of Community and Public Health, Birzeit University, Birzeit, Palestine; 2 Department of Public Health, College of Health Sciences, Qatar University, Doha, Qatar; University of Oslo Faculty of Medicine: Universitetet i Oslo Det medisinske fakultet, NORWAY

## Abstract

Climate change is a critical global risk to humans and ecosystems, influencing water and food security, air quality, and shelter, causing substantial direct and indirect health consequences. This includes heightened risks of infectious diseases, undernutrition, heat-related morbidity and mortality, and a rise in non-communicable diseases (NCDs). Health systems encounter dual challenges: they are highly influenced by the adverse impact of climate change and contribute to climate change through greenhouse gas (GHG) emissions. The effects of climate change on health systems are more severe in low- and middle-income countries, which are the least responsible for global emissions. Palestine’s challenges are intensified by prolonged military occupation, limited resources, and a fragmented health system, making it especially vulnerable. This study explores the current health system and policy response to climate change in the West Bank, Palestine, with the aim of identifying gaps and determining priority actions to strengthen health system resilience and sustainability. This is a descriptive qualitative study that applied the 2023 WHO framework for climate-resilient and low-carbon health systems. The research utilized policy document analysis of climate-related national policies and health-related sector strategies, plans, practices and regulations. This is complemented by key informant interviews (KIIs) with different relevant stakeholders from different institutions and governance levels, and focus group discussions (FGDs) with different stakeholders in the West Bank. Findings reveal that while some polices on climate resilience and sustainability have been developed, their implementation remains limited. The workforce is insufficiently trained, and there is limited routine data and research on climate and health. Moreover, the health system has not yet integrated resilient and sustainable health operations, technologies, and infrastructure into service delivery. Furthermore, financial constraints are a major barrier, as the system operates under budgetary pressure and scarce resources. Priority actions include strengthening climate-related data collection and research, improving workforce capacity through structured training, increasing community awareness, and securing funding for resilient and sustainable health operations. Addressing these issues is fundamental to strengthening the Palestinian health system in response to climate change.

## Introduction

There is strong evidence that climate change is one of the greatest threats facing humanity and ecosystems [1–4]. It negatively influences all life necessities, including air, water, food, energy, shelter, and health [5–7]. Climate change threatens water security [8,9], contributes to food insecurity [10–12], and increases the risk of infectious diseases, undernutrition, heat-related morbidity and mortality [2,13], and non-communicable diseases such as cancer [14], cardiovascular diseases [15], and respiratory diseases [16]. Additionally, climate change and its health impacts place burdens on health systems [17] disrupting their ability to function effectively [18]. At the same time, the health sector is also a contributor to climate change, as its daily operations generate substantial greenhouse gases (GHG) [19,20].

The impact of climate change on human health and health systems is inconsistent and disproportionate across populations and countries [21]. Compared to high-income countries, health systems in low- and middle-income countries (LMICs) and least developed countries (LDCs) experience greater impacts despite contributing least to global emissions [22]. This is due to challenges such as resource scarcity, limited funding, system fragmentation, policy incoherence, underdeveloped health information systems, inadequate human resources, poor inter-sectoral collaboration, and low community participation [22–24].

In addition to these LMIC challenges, Palestine is particularly vulnerable to climate change, with evidence of more frequent and extreme heatwaves, water scarcity, and growing risks of droughts [25,26]. These vulnerabilities, amplified by the prolonged military occupation that controls land and resources, contribute to GHG emissions, and affect the accessibility and continuity of services [27,28]. Moreover, Palestine’s fragmented political landscape and complex legal framework have resulted in a poorly coordinated health system with limited resources. These barriers hinder the health system’s ability to adapt to climate change and mitigate its causes and impacts [29–32].

According to the World Health Organization (WHO) Operational Framework, “climate resilient and low carbon health systems are those capable of anticipating, responding to, coping with, recovering from, and adapting to climate-related shocks and stress, while minimizing GHG emissions and other negative environmental impacts to deliver quality care and protect the health and well-being of present and future generations” [19]. Achieving this requires urgent action in strengthening and improving the system’s building blocks: leadership and governance, health workforce, health information systems, essential medical products and technologies, service delivery, and financing [33–35]. In conflict-affected and resource-constrained settings like the West Bank, this challenge is especially acute.

This study explores the current Palestinian health system and policy response to climate change, with the aim of identifying gaps and determining priority actions to strengthen health system resilience and sustainability under conditions of environmental and political stress.

## Methods

This is descriptive qualitative research that included the analysis of 13 documents, in addition to 15 key informant interviews (KIIs) and 4 focus group discussions (FGDs). As the first study of its kind on climate change and the health system in Palestine, the qualitative approach provides an in-depth understanding of the current situation, practical implementation challenges, and priority actions to enhance the health system’s resilience and sustainability, and provides an evidence-based insights for policy formulation.

The study applied the WHO operational framework for climate-resilient and low-carbon health systems [19] to systematically analyze climate change adaptation and mitigation efforts in the West Bank. Although this framework focuses on health systems, the authors recognize that climate resilience requires a multisectoral perspective. Accordingly, authors augmented the analysis to consider key health-determining sectors. The Environment Quality Authority (EQA), the central authoritative body for all environmental issues in Palestine, identifies 12 priority sectors for climate change adaptation and mitigation, namely agriculture, coastal and marine, energy, food, gender, health, industry, terrestrial ecosystems, tourism, urban and infrastructure, waste and wastewater, and water [36]. These sectors were therefore considered in our analysis to capture interdependencies and inform more integrated policy recommendations.

### Policy document analysis

Authors conducted a desk review to identify any policies, regulatory frameworks, analytical or situational analysis reports, and other documents -hereafter collectively referred to throughout this paper as “policy documents”- related to climate change in Palestine. A combination of methods was used to locate these documents, including online searches and direct contact with stakeholders to access materials unavailable online. Authors developed a document screening checklist by adapting a 5-point scale approach from the scoping review “*Public Health Impact and Health System Preparedness within a Changing Climate in Bangladesh: A Scoping Review*” [37], which itself was inspired by the World Bank’s readiness assessment framework. The checklist was tailored to assess the integration of climate change into each document and its relevance to health and other determinants of health. In order to minimize bias, the checklist was pilot-tested by the research team to ensure consistency. This process identified relevant documents for inclusion in the analysis, enabling a focused review of policy integration. Then, the authors built a comprehensive document review tool to evaluate policy documents for integrating climate change into the existing health system and policy response in the West Bank, Palestine. Using key indicators aligned with the WHO framework, this tool allowed the researchers, to identify strengths, gaps, and opportunities for enhancing climate resilience and sustainability within the health system [19,38,39].

### Key-informant interviews

The research team built on the analysis of the policy documents and the identified gaps and developed interview guides aimed at exploring stakeholders’ perspectives on climate-related policies and health system responses in the West Bank, Palestine. Each guide included open-ended questions with probes, addressing general climate policies, specific health policies, and system-level strategies, plans, and practices for climate adaptation and mitigation; they were refined through two pilot interviews with participants homogenous to the main study population. The guides were built based on the WHO framework, considering the building blocks of the health system and the unique socio-political context of the region [19].

Utilizing a purposive sampling, we selected relevant stakeholders at various levels from different institutions and governance levels, who are actively engaged in building the “climate-resilient and sustainable health system” (see S1 File for detailed information about participants).

Between June and July 2024, the researchers, MA and YW conducted 15 semi-structured KIIs, both face-to-face and through phone calls or Zoom meetings. This number was determined to confirm adequate representation of participants and to achieve data saturation where no new themes were emerging from additional interviews. The interviews, each lasting on average around 45 minutes, were recorded to capture participants’ opinions and views on the current situation, possible solutions, and alternatives to improve the country’s ability to build this response. Verbal consent was obtained on the day of the interview. At the end of each day, researchers listened to the recorded interviews, transcribed them verbatim, wrote notes, and reviewed findings to assess saturation. Each transcript underwent a multi-stage coding process through MAXQDA24 software, resulting in the tree of codes (see S2 File).

While the analysis mainly followed an inductive coding approach [40], where themes and subthemes emerge from the collected data, the structure and the organization of the findings were informed by the WHO framework and its health system building blocks [19]. After the initial open coding, which identified the meaningful segments and subsequently the initial codes, axial coding was performed to examine the relationships between codes and group them into subthemes. Through thematic analysis, these subthemes were organized into broader themes that present the main idea across the dataset. Triangulation was achieved by a combination of data triangulation (by collecting data from various levels of participants) and investigator triangulation (by involving multiple researchers in the data coding and interpretation) to enhance the credibility and consistency of the findings.

### Focus group discussions

To supplement and enrich the interviews, four face-to-face FGDs were conducted on 24 July 2024 with key stakeholders from the health sector and other health determining sectors that are directly engaged in the climate change topic (see S1 File for detailed information about participants). These FGDs gathered collective insights and facilitated an interactive discussion that focused on the current situation in responding to climate change, their understanding, vision, and any experience in responding to this crisis, and their views of the barriers and solutions to planning this response.

Authors utilized a purposive sampling to select the relevant stakeholders from the health sectors and other health-determining sectors. A total of 23 participants took part in four FGDs, three groups had six members each, and one had five. Participants were distributed across groups to ensure balanced diversity in backgrounds, promoting a comprehensive exchange of perspectives. FGDs were facilitated by the author themselves, in addition to trained note-takers, using a semi-structured discussion guide. Verbal consent was obtained from each participant prior to participation. Discussions were audio recorded, in addition to field notes. Data collectors transcribed the FGDs, and then the authors analyzed them using MAXQDA24 software, following the previously described inductive coding process applied to the interviews. Triangulation was applied through multiple coders and the integration of data from both interviews and FGDs.

### Ethical considerations

Ethical approval was granted by the Research Ethics Committee at the Institute of Community and Public Health, Birzeit University (Reference number: 2023 (12-1)). All participants were provided with detailed written and verbal information about the study. Verbal informed consent was obtained from each participant in accordance with the national legislation and the institutional requirements. Confidentiality and anonymity were maintained throughout data collection, storage, and analysis to protect participants’ privacy.

## Results

A total of 13 documents were included in the document review, comprising 6 policy documents, 3 regulatory frameworks, 2 situational analysis reports, and 2 technical consultancy reports (Table 1). Data collected from the policy document review, key-informant interviews, and FGDs were categorized based on the WHO framework into the six domains [19] (see S2 File), reflecting the current situation of the Palestinian health system in terms of response to climate change.

**Table 1:** List of documents included in the analysis.

#	Title	Year	Organization and Author\s	Type
**1**	Initial National Communication Report to the United Nations Framework Convention on Climate Change (UNFCCC) [41]	2016	Environment Quality Authority	Policy document
**2**	National Adaptation Plan (NAP) to Climate Change [36]	2016	Environment Quality Authority (Richard Smithers, Mike Harrison, Ziad Mimi, Khaled Hardan, Sadiq Abdelall, and Afif Hasan)	Policy document
**3**	Occupied Palestinian Territory Health and Climate Change Profile 2022 [42]	2022	World Health Organization, United Nations Framework Convention on Climate Change	Situational analysis document
**4**	The State of Palestine’s First Nationally Determined Contributions (NDCs) [43]	2021	Environment Quality Authority	Policy document
**5**	The State of Palestine’s Nationally Determined Contribution (NDC) implementation plans: Health – Developing safety and monitoring systems for water, food and sanitation [44]	2021	Ricardo Energy & Environment (Munir M. Qazzaz, Martha Preater, Islah Jad, Clémence Moinier and Richard J. Smithers)	Technical consultancy report
**6**	Policy implementation plan to support delivery of the State of Palestine’s NDC implementation action plan for Health – Developing safety and monitoring systems for water, food and sanitation [45]	2022	Ricardo Energy & Environment (Martha Preater, Maya Rubin, Afif, Hasan, Rachael Steller and Richard J. Smithers)	Technical consultancy report
**7**	Framework for mainstreaming health in climate change actions and mainstreaming climate change in the health sector [46]	2022	Environment Quality Authority	Regulatory framework
**8**	Toolkit for Mainstreaming Gender in National Climate Change Plans in Palestine [47]	2020	Environment Quality Authority and UNDP	Regulatory framework
**9**	“Technology Roadmap for the Implementation of Climate Actions Plans in Palestine” Gender Mainstreaming Report [48]	2019	Environment Quality Authority and Climate Technology Centre & Network	Regulatory framework
**10**	Law No. (7) of 1999 Palestinian Environmental Law [49]	1999	State of Palestine	Law
**11**	Decree Law No. (31) Of 2021 AD amending Law No. (7) of 1999 AD regarding the environment [50]	2021	State of Palestine	Decree-Law
**12**	Stakeholder Engagement Plan (SEP) [51]	2023	Palestinian Ministry of Health (MoH)	Policy document
**13**	Sustainable Energy Access & Climate Action Plan (SEACAP) [52]	2023	Ramallah Municipality & Clima-Med experts	Situational analysis document

### Domain 1: Leadership and governance

Leadership and governance establish the foundation for effective climate change responses. This domain presents key aspects including policy development, implementation efforts, and collaboration initiatives, highlighting both progress and existing gaps ensuring that leadership and governance drive planned, coordinated, and impactful climate actions (see S2 File).

#### Policy development.

Findings from the policy document review emphasized Palestine’s efforts to align with the international climate commitments. Palestine has become the 197th party to the United Nations Framework Convention on Climate Change (UNFCCC), as it ratified the Paris Agreement in 2016 [53], and submitted the Initial Communication Report (INCR) initiative [41,54]. Afterwards, in 2016, the EQA released the National Adaptation Plan (NAP) to Climate Change, which covers several aspects, including an evaluation of historical climate trends in Palestine, the identification of highly vulnerable sectors, and the technical aspects required for adaptation [55]. Palestine also developed its first Nationally Determined Contributions (NDCs) in 2017, and updated them in 2021 [43], which prioritize combating climate change nationally through adaptation and mitigation.

In 2021, the Palestinian Authority amended Law No. 7 of 1999 regarding the environment [11], resulting in the enactment of Law No. 31 of 2021 [49,50]. This law mandates climate adaptation and mitigation, requiring all facilities in Palestine to comply or face penalties [50]. It also addresses forming a National Climate Change Committee (NCCC) to follow up on climate change-related conventions [43,49].

In 2022, the EQA developed a framework with an action plan for mainstreaming health in climate change actions [46]. This document identifies cyclical stages, starting with engaging stakeholders and raising awareness, assessing risks, addressing main concerns, and working on mitigation policy options, followed by implementation, then monitoring and evaluation. Furthermore, in 2023, the EQA released a report and a toolkit for mainstreaming gender in climate change national plans and policies [47,48].

Despite progress in developing some climate resilience and sustainability policies, considerable challenges persist in policy formulation and integration. The policy document review revealed that, within the existing political and financial context, climate change is still not prioritized within the national policy framework, often overshadowed by other political, economic, and health concerns. These findings were further highlighted by participants in the FGDs, who stated the limited attention of climate change in the national agendas. In addition, participants pointed to a disruption of policy continuity related to a shifting government agenda. As one senior male stakeholder said:

*“Government priorities change with every new administration, which makes it difficult to sustain a long-term strategy, or secure consistent funding for climate actions This is exactly what happened in our institution, climate change was included in the suggested strategy, but once the administration changed, it was deprioritized”.*

Additionally, like in many other countries, Palestine’s health sector remains under-represented in climate adaptation and mitigation policy initiatives. The absence of strong leadership in health-focused climate governance undermines the health sector’s response to climate change. This concern was raised in the KIIs, where a female department head emphasized:

*“Authorities need to be made aware of the impact of climate change on health so that this issue would be a priority to them.”*

#### Implementation.

The EQA issued policy implementation plans [44,45] setting out logistic preparations such as timeframes, expected costs, and suggested multi-ministerial committee arrangements. However, participants in the KII and FGDs argued that plans should be translated into actions, as most climate-related policies exist only on paper because of the lack of clear enforcement mechanisms in all relevant sectors. Some mentioned examples, including air quality standards and policies promoting electric vehicles, which exist but are neither effectively supported nor enforced.

Adaptation and mitigation efforts are not systematically embedded within the national health program, as initiatives implemented in response to climate change are mainly reactive rather than the result of structured planning. Moreover, participants highlighted enforcement challenges, particularly regarding the monitoring and management of medical waste, which stakeholders repeatedly emphasized, as stakeholders in the KIIs and FGDs noted:

*“There are policies, but the issue lies in enforcement.”*

Additionally, donor-driven priorities make external funding dictate projects without fully integrating them into a unified national framework, as stakeholders in the FGDs highlighted:

*“Donor-driven priorities influence the implementation phase, leading to fragmented strategies that do not align with national policies.”*

#### Collaboration.

Participants in the KIIs identified the EQA as playing a central role in facilitating collaboration among sectors. It works closely with the MoH, Ministry of Agriculture, Ministry of Transport and Communications, and other governmental bodies to address climate actions. At the national level, participants pointed out that the NCCC, which consists of officially appointed members from all relevant sectors, is considered the central body for coordinating climate-related efforts, tasked with multi-sectoral agreements and collaborations. Despite these structures, FGDs participants noted that challenges persist in ensuring effective cross-sectoral implementation due to unclear roles and responsibilities, duplication of efforts, and limited resources. Translating these agreements into implementable actions remains challenging.

Shared activities among sectors were identified; for example, the Ministry of Agriculture collaborates with the Palestinian Meteorological Department, under the supervision of the EQA, in a project that supports smart agriculture to adapt and mitigate climate change. Furthermore, several joint efforts between the Ministry of Agriculture and the Water Authority were mentioned. However, different stakeholders from various sectors in the KIIs pointed out limited collaboration with the health sector. One active member of the NCCC mentioned:

*“No, there are no joint programs or initiatives between us and them. In fact, the health sector is the sector with the least participation between us and them.”*

To strengthen the collaboration and increase the involvement of certain sectors, participants in the FGDs suggested establishing formalized communication channels between health, environment, and other relevant sectors. In addition, participants mentioned that climate focal points already exist within each priority sector and serve as primary contacts for coordinating and implementing climate actions.

### Domain 2: Workforce

The workforce plays an essential role in the effectiveness of climate adaptation and mitigation measures. Our findings highlighted factors related to individual and organizational capacity, as well as communication practices, that influence the effectiveness of climate change response (see S2 File).

#### Individuals’ capacity building.

Capacity building is one of the activities included in the NDCs implementation action plans [44,45]. In another document, the Sustainable Energy Access and Climate Action Plan [52], strategic adaptation measures, emphasizing women’s empowerment and capacity building were highlighted. Participants in the KIIs mentioned a few existing training initiatives on climate change; most of them are conducted as single-day workshops. The content of these initiatives covers general topics, including general environmental awareness. The EQA mentioned a workshop for training members of the NCCC on how to access international climate funds. However, there is no specialized training on climate change response in priority sectors, including the health sector, nor is there a curriculum.

In terms of the health workforce, participants were concerned about insufficient knowledge on climate change among healthcare providers and front liners, as well as their limited ability to address and respond to climate-related health consequences. As one participant who engages with healthcare professional in the KIIs put it:

*“The vast majority of healthcare workers do not know the basics of dealing with various medical wastes within their health facilities, even before they are released for final disposal”*

Furthermore, participants in both KIIs and FGDs criticized the absence of climate-related content across all levels of education curricula, which leaves the workforce underprepared and affects the improvement of solid expertise within sectors.

#### Organizational capacity.

Beyond the individual capacity, the organizational capacity of all priority sector institutions is important. Excessive workload placed on a limited number of workforces in some departments related to environmental work was a repeatedly mentioned challenge affecting the prioritizing of climate change work. A senior female participant in the KIIs expressed:

*“We handle numerous environmental responsibilities with a limited staff, which leaves us overburdened.”*

#### Communication.

Country-specific health and climate change profile for the occupied Palestinian territories (2022), by WHO and UNFCCC [42], includes guidance on how to enhance public awareness and guide decision-makers in taking targeted action. Despite this, participants in both KIIs and FGDs identified limited communication efforts, insufficient public awareness, and inadequate community engagement as major barriers to effective climate change adaptation. One stakeholder in the KIIs, a researcher and a community activist said:

*“Public engagement on climate-related health risks is minimal. Without community involvement, adaptation efforts will remain ineffective.”*

Some sectors have initiated awareness and community engagement activities. The Ministry of Agriculture, for example, provides farmers with awareness of climate-related risks, such as rising temperatures, water scarcity, and pest outbreaks. Similarly, environmental and health organizations are involved in community outreach efforts, although these remain limited in scope and coordination.

Participants in the FGDs emphasized community awareness as critical. They highlighted that raising public awareness is fundamental to the success of environmental and health interventions, as it empowers individuals to take preventive and adaptive measures. However, stakeholders noted a significant gap in structured communication strategies, which limits the effectiveness of awareness initiatives.

Public skepticism remains a challenge in climate communication despite ongoing awareness efforts. However, as awareness grew, people began seeking information themselves, one participant in the KIIs explained:

*“At first, we used to go to people and talk to them repeatedly about climate change, but no one was convinced. Now, they are the ones coming to us.”*

One key aspect of communication that emerged in FGDs was its role in emergency response. The stakeholders explained that public information campaigns are often launched in reaction to extreme weather events, but there is a pressing need for a proactive and sustained communication strategy to ensure community preparedness.

Additionally, some participants discussed the role of media in disseminating climate-related information. While media outlets occasionally provide weather-related updates, their potential as a tool for broader climate awareness remains largely untapped. Strengthening the collaboration between media channels and relevant institutions could enhance public access to critical information and foster greater engagement with climate issues.

### Domain 3: Data collected related to climate change

Data collection and assessment are paramount in understanding climate-related risks, vulnerabilities, and gaps and supporting evidence-based promising opportunities. This domain highlights critical dimensions in climate-related data collection and utilization, including climate risk assessment, environmental impact evaluation, and research efforts (see S2 File).

#### Assessment of climate risks and GHG emissions.

Understanding climate change-related risks for priority sectors is fundamental for addressing adaptation efforts, while examining GHGs is important in shaping mitigation efforts. The NAP and the NDCs present some climate-related risks; however, this information is based on reviewing future projections submitted to the UNFCCC by neighboring countries such as Egypt, Jordan, and Lebanon. Additionally, it is based on unknown current or future local levels of GHG. This reliance on data from external sources emphasizes the need to invest in country-specific climate data.

Participants in the KIIs indicated that the priority sectors acknowledge the climate-related risks with varying degrees of understanding of their size. The health sector recognizes climate-related health risks and associated health concerns, such as heat stress and vector-borne diseases; however, these risks are often understood broadly, and addressed in isolation from adaptation efforts, hindering the implementation of targeted interventions. For example, despite the acknowledgement of heat stress as a growing concern, there is no heat preparedness through a heat-health early warning system or planning to reduce the exposure among vulnerable groups.

Regarding GHG emissions, stakeholders in the FGDs expressed that the country assessment does not rely on real-time measurements but estimations using mathematical models and baseline indicators, such as the number of vehicles, which affect the national emissions inventories and, accordingly, mitigation efforts. Of the priority sectors, the health sector’s emissions are rarely integrated into mitigation efforts despite their contribution to climate change through their energy consumption, medical waste, and other activities.

#### Environmental impact assessment (EIA).

In the Palestinian context, findings from policy document review, KIIs and FGDs revealed that there is no specific data collection procedure that is designated to address climate change and its related indicators. However, the EIA process that aims to evaluate and anticipate the potential environmental effects of any proposed development could be considered an important regulatory mechanism. This process is guided by standardized environmental quality criteria, although these criteria are not explicitly climate-focused; it addresses other fundamental environmental dimensions that are strongly related to climate resilience and sustainability.

Participants in the KIIs shared that while some inspections are conducted by the EQA, enforcement of the standardized criteria across sectors remains weak. A participant said:

*“There are standards set by the Palestine Standards Institution regarding air quality and gas emissions, defining permissible limits in Palestine, but implementation is weak.”*

Concerning the health sector, participants in both the KIIs and FGDs discussed the absence of standardized criteria for environmental quality specific to the health sector and for key environmental determinants of health, such as air and water quality, within healthcare facilities and the surrounding environment.

#### Research.

Climate change is a relatively new topic for research in Palestine, and it is still underdeveloped. Participants in the FGDs indicated that climate-related research efforts, encompassing master’s theses, doctoral dissertations, and institutional studies, rarely addressed climate change and health. Moreover, they emphasized the lack of a research agenda. Many stakeholders underscored the necessity for a national research agenda developed through a needs assessment of vulnerable people and sectors, rather than dictated by donor priorities. One stakeholder in the FGDs said:

*“Who are the most affected? Often, it is the poor and marginalized communities, including the elderly, children, youth, women, and small-scale farmers—those we need to consider.”*

On the other hand, they noted that the country’s research efforts lack comprehensive assessments. Although certain entities collect domain-specific data, information remains unintegrated and poorly disseminated across sectors. However, participants also added that the EQA is developing a national climate platform to bring together the different engaged parties and their efforts to improve data accessibility and enhance integration and communication.

### Domain 4: Essential medical products and technologies

This domain covers efforts to integrate climate-friendly practices within the Palestinian health system’s products and technology (see S2 File).

#### Sustainability of the health operations.

Health sector stakeholders in both the KIIs and FGDs highlighted that the Palestinian MoH confirmed its commitment to climate change resilience and mitigation during the UN Climate Change Conference in 2021 (COP26) by decreasing the carbon footprint and emissions associated with health operations. Nonetheless, this commitment has yet to be implemented, as no particular action plans have been formulated.

On the other hand, some initiatives for improving environmental health in general were initiated, including smoking bans in health centers, increasing green spaces, using solar energy and others, as a stakeholder from the health sector in the KIIs argued:

*“Municipal facilities have transitioned to energy-saving lighting, and new buildings are equipped with sensors that automatically turn off lights in unoccupied areas. We promoted electric vehicles and sold all our cars replacing them with electric ones.”*

Other activities were executed at clinics, mainly aiming to effectively manage waste and improve energy consumption patterns, another health sector participant in the FGDs added:

*“We have begun implementing new projects in some clinics and hospitals, such as energy production using solar panels and waste management initiatives.”*

Talking about the health-determining sectors, participants pointed to some progress in terms of planning and implementation of mitigation initiatives, including the agriculture sector, in which some mitigation programs have been implemented in collaboration with the Environmental Quality Authority.

Stakeholders in the FGDs emphasized that the sustainability of the system should be considered in the early stages of planning, taking into consideration the energy sources and consumption, the design of healthcare-providing buildings, and waste management. For example, one key high-level managerial participant discussed:

*“We need to think about the environmental elements: air, water, food, and soil, to consider the carbon footprint and see how we can save energy in hospitals... One of the most important interventions is how we legally require all healthcare facilities to properly dispose and treat medical waste, knowing that there is a system, but it’s not being implemented properly.”*

#### Infrastructure and technology.

The use of advanced technologies is another activity included in the NDCs implementation action plans. However, stakeholders in all sectors in the FGDs, including the health sector, emphasized that there is a lack in the technological requirements of many possible initiatives for adaptation and mitigation, which could be due to the lack of resources or knowledge. It was also attributed to weak infrastructure, that often, does not support advanced technologies. Additionally, the weak infrastructure, such as improper insulation and old design of buildings with limited heating and cooling systems, and traditional sources of energy, makes it difficult to implement adaptation within the health facilities. Through the KIIs and the FGDs, many stakeholders agreed that to strengthen the health system and increase resilience, there should be an increased investment in enhancing infrastructure.

### Domain 5: Service delivery

The findings highlight key dimensions of service delivery in the Palestinian health system’s response to climate change. For this domain, authors consider service delivery in terms of adaptation and mitigation efforts and barriers that continue to affect effective, resilient, and sustainable service delivery (see S2 File).

#### Adaptation.

The health system response priority is adaptation, and it is primarily focused on responding to climate-related health risks like vector-borne diseases; however, this response remains mainly reactive rather than proactive, with limited specialized climate-health initiatives. As a senior policymaker from the health sector in the KIIs noted:

*“Our priority is adaptation, and in the health sector, adaptation is perhaps most evident in dealing with outbreaks and health emergencies resulting from climate change consequences, when occur, we confront them with all possible efforts.”*

In addition, participants in both the KIIs and the FGDs emphasized the initiation of introducing renewable energy solutions such as solar panels in some healthcare facilities to reduce dependence on unstable electricity sources from climate-change-related fluctuations in water availability.

#### Mitigation.

Regarding mitigation, KIIs and FGDs revealed that GHG reduction measures remain underdeveloped, with limited recognition for reducing the health system footprint. Participants expressed concerns about medical waste management, as improper disposal measures lead to marked environmental and public health threats. Although some facilities have implemented basic waste segregation, there is no proper final disposal system. A participant who engages with health care professionals in the KIIs said:

*“Medical waste is a primary output of healthcare services. What happens to it? It is separated in health centers in sharp boxes, then how is it dealt with? Unfortunately, we do not have a protocol for proper disposal.”*

On the other hand, the use of renewable energy at small scales and the introduction of electric vehicles in some facilities were also mentioned as attempts to reduce reliance on fossil fuels to produce energy.

#### Challenges of implementation.

Documents revealed that the implementation of both adaptation and mitigation measures is hindered by financial constraints, the political instability in the region, and the structural barriers. Participants brought into focus the main reliance on external funding, with inadequate national budget allocation for climate-health initiatives, which limits the ability to invest in climate-resilient and sustainable infrastructure and service delivery. One stakeholder involves in fund securing added:

*“As a country, that depends mainly on external funding. If I want to introduce strategies to withstand, for example, heat waves, like providing more water and more electricity, I am facing a financial challenge. I cannot provide this amount from project allocations.”*

Participants in the FGDs discussed that political challenges, including resource scarcity and restrictions on importing environmentally friendly technologies, exacerbate implementation difficulties. Pre-existing challenges—being overburdened, unstructured, and fragile—hinder the system’s capacity to incorporate resilient and sustainable measures into service delivery.

### Domain 6: Financing

National budgets and international aid are essential to support climate change response and protect people’s health. The complexity of climate change impacts requires that authorities be prepared to respond timely and properly by implementing methods that facilitate the availability, allocation, and utilization of financial resources. In this domain, authors present current efforts, challenges, and needed future actions to strengthen the climate financing within the health sector (see S2 File).

#### National budget for climate change work.

The NDCs document includes a planned budget for highly vulnerable sectors, including the health sector, but it is not specific and clear. Like many other LMICs, Palestine has no allocated national budget for adaptation and mitigation actions. Participants in the FGDs reported that many projects and action plans have not started yet because of the lack of financial resources. This applies to the health sectors and the health-determining sectors. As some stakeholders mentioned:

*“There is still a funding gap. The financing is fragmented and insufficient to meet the actual needs. If adequate local or national funding were available, we would have established a dedicated climate change department with staff, governance systems, and infrastructure—but currently, that remains a distant goal.”*

In addition, stakeholders in the KIIs expressed the lack of national allocated budgets for emergencies, which leads to a delay in appropriate responses, for example in cases of heavy rain or droughts. As one participant said:

*“We need to have budgets ready in case of emergency, when you have an early warning system in place you have to have a budget so that if a problem occurs, I can intervene before it happens and be prepared … but unfortunately we don’t have it.”*

#### International aid.

The NDC’s action plans aim to provide practical guidance to facilitate implementation and increase the chances of receiving funds. The WHO Health and Climate Change Global Survey reported that the level of implementation of the health and climate change action plan is low in Palestine [42], which could be supported through the exploration of further opportunities to access funds that prioritize health and climate change [54]. Participants, especially those from EQA and the MoH, affirmed this. They mentioned that the climate action plans included estimations of the needed financial resources for each plan to be implemented and that they need proper training and guidance to secure and access international funding. As one stakeholder from the EQA in the KIIs explained: *“These 14 plans, their cost is six billion dollars! It’s not an easy thing. It’s a priority, meaning a priority for six sectors at the national level. Our role is to work with these ministries, to bring in funding and do fundraising for these plans, to implement them on the ground. We have very good climate planning, but we need financial resources to implement.”*

On the other hand, participants in the KIIs mentioned MoH received funding to implement the preparedness project, which was described as an initial project to define the local needs to tackle the climate crisis and its expected future challenges. This project, if implemented appropriately, will enable them to apply for larger funding, such as the Green Climate Fund.

## Discussion

This study is the first analysis of the health system and policy response to climate change in the West Bank, Palestine, utilizing the WHO Operational Framework for building climate-resilient and low-carbon health systems as a guiding framework [19]. Utilizing document analysis, KIIs, and FGDs, key findings identified strengths, gaps, and priority actions necessary to enhance the resilience and sustainability of the Palestinian health system amid the climate change crisis.

While Palestine demonstrates a great national and international commitment to addressing climate change through policy development initiatives like the NAP and the NDCs, there is still a lack of a clear, orientated, and integrated policy framework that links climate action to health sector planning. Additionally, its programs and activities remain ad hoc, not based on plans or priorities. However, Palestine is no exception; globally, only 30% of the 83 countries that committed to climate action at COP26 had developed a national health and climate change plan or strategy [35]. This is related to many factors, including health systems’ primary focus on immediate health concerns, such as communicable and non-communicable diseases, leaving public health threats like climate change as a secondary priority. In addition, like many other LMICs [56], the prevailing climate policy development approach is sector-specific, where environmental authorities often direct it. At the same time, health sector planning is managed independently [57]. This suboptimal coordination in planning between health and other health-determining sectors affects the representative integration of climate action into health policies.

Frequent leadership changes and unclear stakeholders’ roles and responsibilities for climate and health were identified as obstacles to proper response and coordinated efforts. Global research indicated the importance of empowering stakeholders with clear roles, especially those at the middle management level, in building resilience [34], as they are thought to be important role players close to frontline workers who could support and enhance response initiatives.

Additionally, Findings reveal several critical gaps in Palestine’s health system and policy response to climate change. Key challenges include limited workforce capacity and insufficient climate-specific trainings, weak community engagement, inadequate data collection and reliance on external sources, gaps in regulatory enforcement, underdeveloped climate-health research, insufficient integration of resilient and sustainable technologies into service delivery, and financial and political constraints that limit effective climate actions.

For the workforce, strengthening capacity through knowledge, awareness, and proper skills related to the additional burdens posed by the climate crisis is an essential component of an overall resilient health system [19,58]. This is particularly important in light of evidence showing limited climate-specific health literacy among health professionals, which may hinder their ability to address climate-related health issues effectively [59]. Existing training initiatives are often short-term, with minimum depth and structure, and do not target specialized climate-related competencies, especially among the healthcare workforce. The absence of structured climate change training curricula within both formal education and professional training limits the workforce’s capacity to respond. Global studies indicate a need to have well-structured health professionals’ education that focuses on the health consequences of climate change [60], and how to treat climate-related diseases [56], across education levels. A case in point is Azerbaijan, where the health authorities, in partnership with the UNICEF, initiated the integration of climate change and environmental health topics into the existing national curriculum for healthcare professionals at key educational institutions [61]. Additionally, inter-professional education could provide a unique opportunity for enhancing health workforce capacity, which still suffers from limited implementation globally [60].

Findings highlight a critical gap in institutional commitment to climate responses due to insufficient human resources allocation. Overburdened staff and resource constraints weaken the integration of climate-related tasks into routine operations. One key aspect that emerged from the findings is inadequate community engagement. Communities may help participating in planning, implementation, and monitoring [56]. To address local concerns, tailored and integrated response plans require community consultation, including participation from indigenous, vulnerable, and marginalized communities, which will improve healthcare access and quality [21,62]. Such successful engagement can be seen in Ethiopia during the response to the most severe weather condition over the past 30 years, the El Niño drought of 2015–2016. A community-based health system that includes organized community groups linked to the health extension workers (HEWs) demonstrated a successful and timely response to drought [63]. In a resource-constrained and politically unstable context like Palestine, there should be greater commitment to creating proper communication channels that foster public awareness and engagement [62], including participatory approaches to engage local communities in climate and health decision-making, building trusted community networks, and the involvement of marginalized and vulnerable groups, including youth and women [64].

Data is essential for decision-making, evidence-based planning, prioritizing efforts, and monitoring progress [65,66]. Data collection is critical in designing and implementing responses [42,67]. It helps policymakers assess risks, identify vulnerable populations, and understand climate change’s environmental, social, economic, and health impacts [66,68]. For adaptation, data helps map climate vulnerabilities, such as extreme weather events, water scarcity, or health risks and guides the development of targeted strategies to reduce exposure and build resilience [42,69]. For mitigation, accurate data on GHG emissions, energy consumption, land use, and pollution levels enable stakeholders to prioritize actions that reduce emissions and improve environmental sustainability [70,71]. Moreover, data is a key to prioritizing efforts, identifying which sectors, regions, or communities require urgent attention. It supports the efficient allocation of resources, ensuring interventions are cost-effective and impactful. Monitoring progress relies heavily on continuous data collection [68]. Without reliable data streams, it becomes difficult to track the effectiveness of policies and actions, adjust strategies, or demonstrate accountability to stakeholders and the public [42,68].

An additional issue is a heavy reliance on external data, such as regional climate projections from neighbouring countries, due to the absence of robust, localized climate risk assessments and GHG inventories [44]. This reliance compromises the ability to tailor national adaptation and mitigation strategies to Palestine’s local and unique environmental and socio-political context. Without country-specific data on current and projected risks and local GHG levels, efforts to design effective and targeted interventions, particularly for high-risk sectors, remain fragmented and reactive rather than anticipatory [70,71].

The findings also highlight gaps in regulatory enforcement. Although Palestine has established environmental standards, such as air quality and emissions thresholds [49,72], weak implementation undermines environmental monitoring efforts. This regulatory gap is particularly concerning for the health sector, where the absence of standardized environmental quality criteria for healthcare facilities poses risks to patient and community health [73,74].

Additionally, the underdevelopment of climate-related research, particularly at the intersection of climate change and health, presents a missed opportunity to inform appropriate policies and practices [75]. The absence of a national research agenda and fragmented and donor-driven research priorities constrains the generation of context-specific evidence. This weakens the ability to identify vulnerable populations and sectors and their specific needs for planned interventions.

Findings showed that the health system has not yet fully integrated resilient and sustainable health operations, technologies, and infrastructure into service delivery, despite commitments. The gap between commitment and implementation [42] can be attributed to weak governance defined by the absence of concrete action plans, lack of systematic monitoring mechanisms, and insufficient enforcement of sustainability policies. The Palestinian case reflects the broader worldwide picture, as only 8% of the countries committed to sustainable, low-carbon health systems have developed action plans after their commitment [35]. Additionally, financial constraints are a fundamental barrier, as the system operates under budgetary pressure and scarce resources [44]. Urgent medical needs take priority over sustainability initiatives. This is consistent with the worldwide situation; of the countries that reported on funding, only 9% reported having adequate funding available to fully implement sustainability into service delivery and technology [7].

These governance and financial barriers are compounded by political restrictions, including limited access to resources and import restrictions that affect obtaining advanced environmentally friendly technologies and adopting sustainable energy solutions [28]. Moreover, like other developing countries [34], weak infrastructure poses adaptive challenges and makes introducing climate-smart modifications costly and complex. Conversely, health infrastructure is more advanced across developed countries like the United Kingdom, Germany, and Japan, incorporating climate-resilient construction and innovative technologies that enhance service delivery resilience and sustainability [31].

The Palestinian health system suffers from massive financial constraints, with no allocated national budget for climate resilience and sustainability actions. Instead, it is severely dependent on international funding, which is challenging and competitive and requires extensive efforts to be secured. Many other countries face the same reality; according to the WHO Global Health Expenditure Database, less than half of the 2020 health expenditures of countries committed to the COP26 Health Program came from the domestic central government [76]. However, despite the fundamental role of external funding, 49% of LMICs reported having access to climate funds from international bodies such as the Green Climate Fund, emphasizing the challenging and complicated climate financing mechanism [57].

Literature has found that reliance on external funds results in a short-term project-based approach, which limits continuity and integration into national policies and actions [7]. Additionally, external funding means priorities often originate from the donor agenda rather than local needs and vulnerabilities [35]. Even if funding is secured, the lack of structured and strategic financial planning further complicates its effective utilization, impacting the efficiency of climate action measures.

### Recommendations

Based on the findings, the authors recommend priority actions to enhance the Palestinian health system’s response and resilience. The most important is enhancing local data collection through surveillance and research establishing a centralized data repository [42,67]. The availability of reliable, context-specific data is the first step toward understanding and predicting the current and future health impacts of climate change [7]. This could be achieved by improving comprehensive real-time surveillance [34], developing a thorough health impact assessment framework to evaluate these impacts [77], and investing in climate-health modelling that allows for better forecasting. All of these help the health system to anticipate climate-related health impacts, identify vulnerabilities, enhance emergency preparedness, and formulate targeted, evidence-based policies that support the system’s resilience and sustainability [65,66].

Considering financial constraints, securing funding and mobilizing resources for climate resilience and sustainability is another top priority [19]. This could be facilitated by strengthening access to international funding mechanisms [20]. This should go hand in hand with ensuring strategic financial planning to ensure obtained climate finance reaches the health system.

Personal and institutional capacity building is another important priority [60]. This involves workforce training through structured programs that integrate climate change into on-the-job training, sectoral curricula, and professional education in the health and health-determining sectors [34]. Additionally, adopting a comprehensive emergency management approach is important. This includes the development of emergency preparedness and response plans with reliable monitoring and evaluation mechanisms [19]. Related to this, it is important to conduct an infrastructure vulnerability assessment and implement strategic retrofitting of healthcare facilities to enhance service delivery resilience and sustainability [56]. Finally, developing public awareness campaigns is essential to engage the community and inform it about climate-health consequences, which enhances climate-conscious behavior [78].

### Strengths and limitations

This study has several strengths, including being the first study to systematically explore the health system and policy response to climate change in Palestine, addressing an important gap in the literature. The context-specific focus ensures the high relevance of the findings and interpretations for informing national policies and plans. In addition, the study design that used the qualitative approach of multiple data sources allowed for in-depth exploration and understanding and enhanced credibility through triangulation.

However, the study does have some limitations. While the context-specific focus allowed depth and relevance and may offer lessons for other LMICs, it may limit the full generalizability of findings to other settings. In addition, as the study explores the situation at a specific point in time, and the climate impacts and the policy processes continue to evolve, findings may shift in the future. Moreover, as health system research touches governance, financing and accountability, participants may frame their views in the desirable institutional and political expectations, which may lead to a social desirability bias. Finally, while the authors did their best to capture every cultural meaning during the translation from Arabic to English when analyzing and reporting, there is still a possibility of losing some nuance in translation.

## Conclusion

This study explored current climate change strategies and initiatives in the West Bank, Palestine, with the aim of identifying existing gaps and priority actions. The findings indicate that, while there has been progress in policy development related to climate resilience, implementation remains limited. The health workforce lacks sufficient training, and there are notable shortages of routine data and research addressing the climate-health nexus. Furthermore, climate-resilient operations, technologies, and infrastructure have yet to be integrated into health service delivery. Financial constraints pose a significant challenge, as the system continues to function under severe budgetary pressures and limited resources.

The findings of this study contribute valuable insights to the global discourse on climate and health, particularly from the perspective of conflict or politically and economically constrained settings. By highlighting systemic challenges, this research underscores the need for context-specific strategies that account for political instability, occupation, and chronic resource shortages. It broadens the global understanding of how climate resilience in health systems must be approached differently in fragile and conflict-affected regions.

The study also points to a pressing need to expand the climate and health research agenda in Palestine and similar contexts. Future research should prioritize longitudinal studies to track health outcomes related to climate change, examine the intersection of social vulnerability and environmental risks, and explore culturally appropriate adaptation measures. Moreover, interdisciplinary collaborations and knowledge exchange should be strengthened to build a more inclusive evidence base. Regionally and globally, the study calls for a shift toward more equity-focused research agendas that center the experiences of marginalized populations living under environmental and geopolitical stressors.

## Supporting information

S1 File List of participants. (PDF)

S2 File Tree of codes. (PDF)
