# Supplementary material for: Health system and policy response to climate change in the West Bank, Palestine: Current situation and priority actions
Source: PLOS Glob Public Health. 2025 Dec 8;5(12):e0005617. doi: 10.1371/journal.pgph.0005617 (PMC12685188; doi:10.1371/journal.pgph.0005617)
Supplement: S1 File — (PDF) [file pgph.0005617.s001.pdf]

Health System and Policy Response to Climate Change in the West Bank, Palestine: Current Situation and Priority Actions

Supplementary Material-1:

List of Participants in Interviews and Focus Group Discussions

	Age Category	Sex	Category of Stakeholder	Organization	Category of Organization	Participation Type
1	50 – 59	F	Healthcare provider	Ministry of Health	Governmental	Interview & FGD
2	50 – 59	M	Upper Management	Ministry of Health	Governmental	Interview & FGD
3	50 – 59	M	Upper Management	Ministry of Health	Governmental	Interview & FGD
4	60 and above	M	Upper Management	Ministry of Health	Governmental	Interview
5	50 – 59	F	Policymaker	Ministry of Agriculture	Governmental	Interview
6	30 – 39	F	Section\department Head	Environmental Quality Authority	Governmental	Interview
7	30 – 39	F	Technical Staff	Environmental Quality Authority	Governmental	Interview & FGD
8	40 – 49	M	Policymaker	Ministry of Transportation	Governmental	Interview
9	30 – 39	F	Technical Staff	Palestinian Central Bureau of Statistics	Governmental	Interview
10	30 – 39	M	Section\department Head	Palestinian Central Bureau of Statistics	Governmental	Interview
11	50 – 59	F	Upper Management	Water Authority	Governmental	Interview
12	-	F	Technical Staff	Ministry of Women's Affairs	Governmental	FGD
13	-	F	Upper Management	Ministry of Local Government	Governmental	FGD
14	-	F	Section\department Head	Ministry of Local Government	Governmental	FGD
15	-	M	Upper Management	Ministry of Local Government	Governmental	FGD
16	-	M	Upper Management	Palestinian Meteorological Department	Governmental	FGD
17	-	F	Section\department Head	Palestinian Meteorological Department	Governmental	FGD
18	-	M	Section\department Head	Palestinian Civil Police Force	Governmental	FGD
19	30 – 39	F	Upper Management	Ramallah Municipality	Quasi-governmental	Interview
20	-	F	Section\department Head	Al-Bireh Municipality	Quasi-governmental	FGD

21	-	M	Section\department Head	Al-Bireh Municipality	Quasi-governmental	FGD
22	-	M	Section\department Head	Hebron Municipality	Quasi-governmental	FGD
23	-	M	Technical Staff	Hebron Municipality	Quasi-governmental	FGD
24	-	M	Upper Management	National Park	Quasi-governmental	FGD
25	50 – 59	M	Researcher	Birzeit University	Academic	Interview
26	-	M	Researcher	Birzeit University	Academic	FGD
27	-	M	Researcher	Al-Quds University	Academic	FGD
28	-	M	Researcher	(Expert)	Academic	FGD
29	-	F	Environmental Expert	(Expert)	Academic	FGD
30	20 – 29	F	Healthcare Provider	Palestinian Medical Relief Society	NGO	Interview
31	60 and above	M	Policymaker	Palestinian Hydrologists Group	NGO	Interview
32	-	M	Programs Director	Action Aid	NGO	FGD
33	-	M	Project Manager	Food and Agriculture Organization	NGO	FGD
34	-	M	Project Manager	Food and Agriculture Organization	NGO	FGD
