# Supplementary material for: Health system and policy response to climate change in the West Bank, Palestine: Current situation and priority actions
Source: PLOS Glob Public Health. 2025 Dec 8;5(12):e0005617. doi: 10.1371/journal.pgph.0005617 (PMC12685188; doi:10.1371/journal.pgph.0005617)
Supplement: S2 File — (PDF) [file pgph.0005617.s002.pdf]

The Current Situation of the Health System in Response to Climate Change in Palestine: Tree of Codes

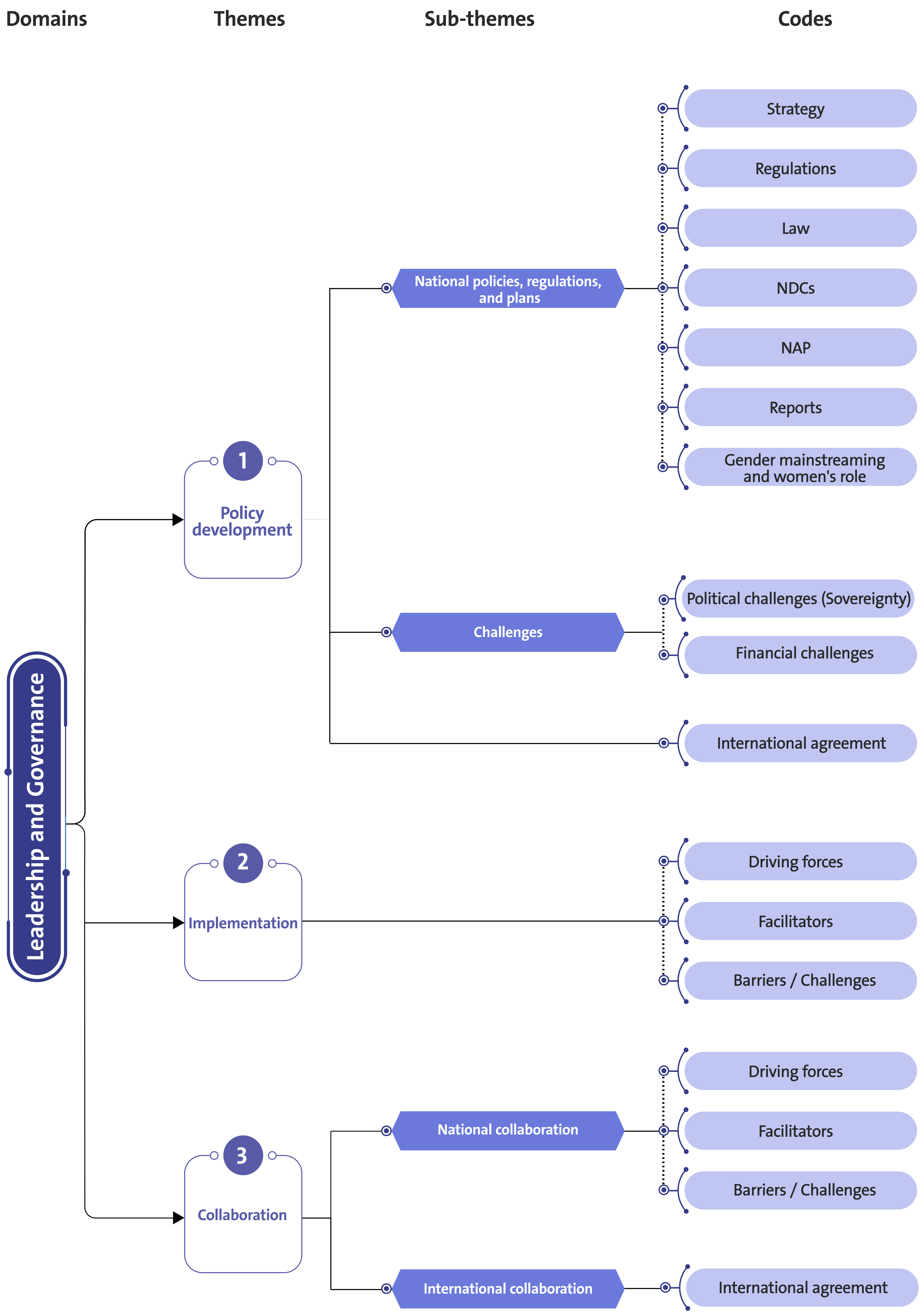

©2025 ICPH-BZU

The Current Situation of the Health System in Response to Climate Change in Palestine: Tree of Codes

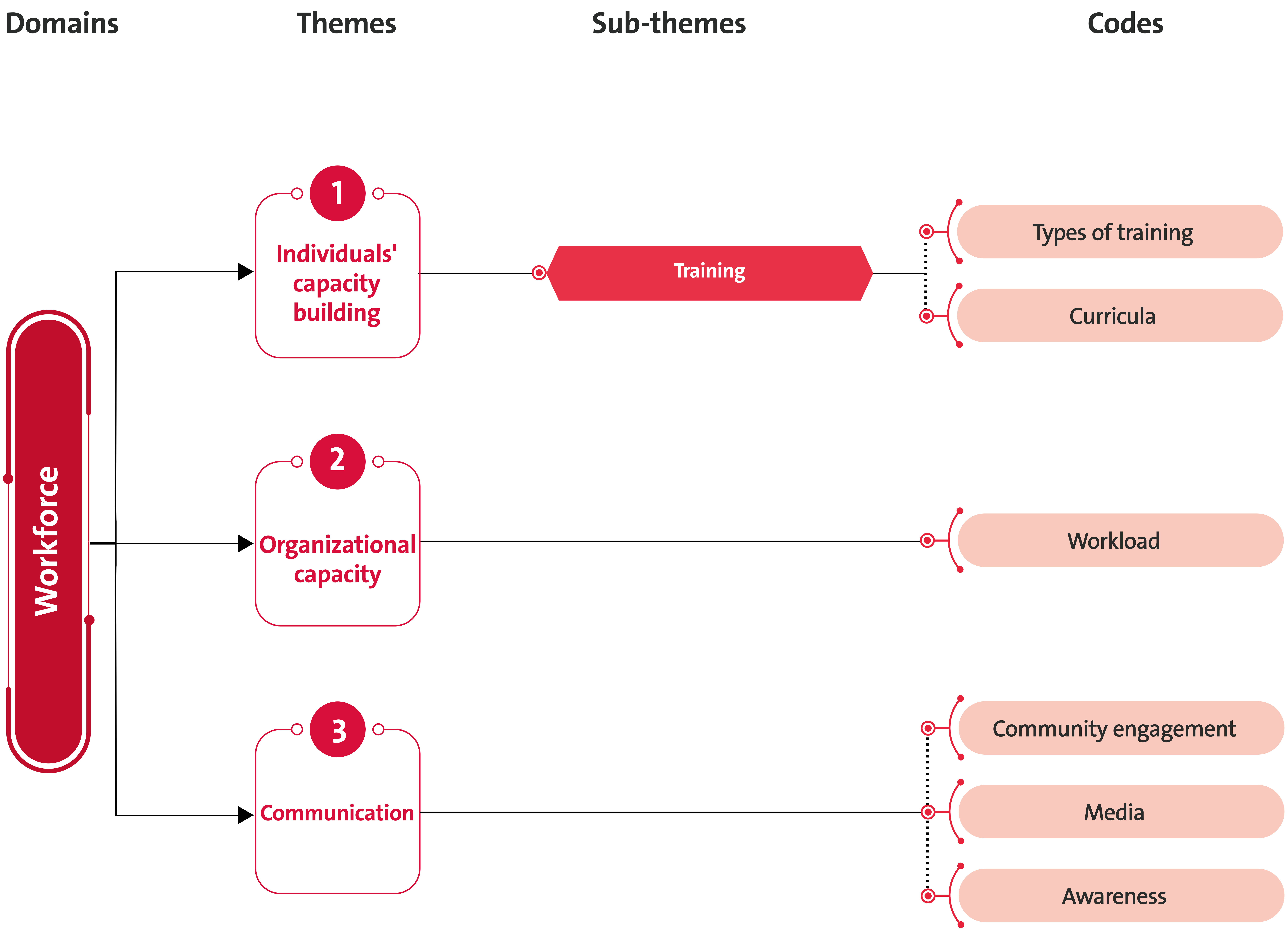

©2025 ICPH-BZU

The Current Situation of the Health System in Response to Climate Change in Palestine: Tree of Codes

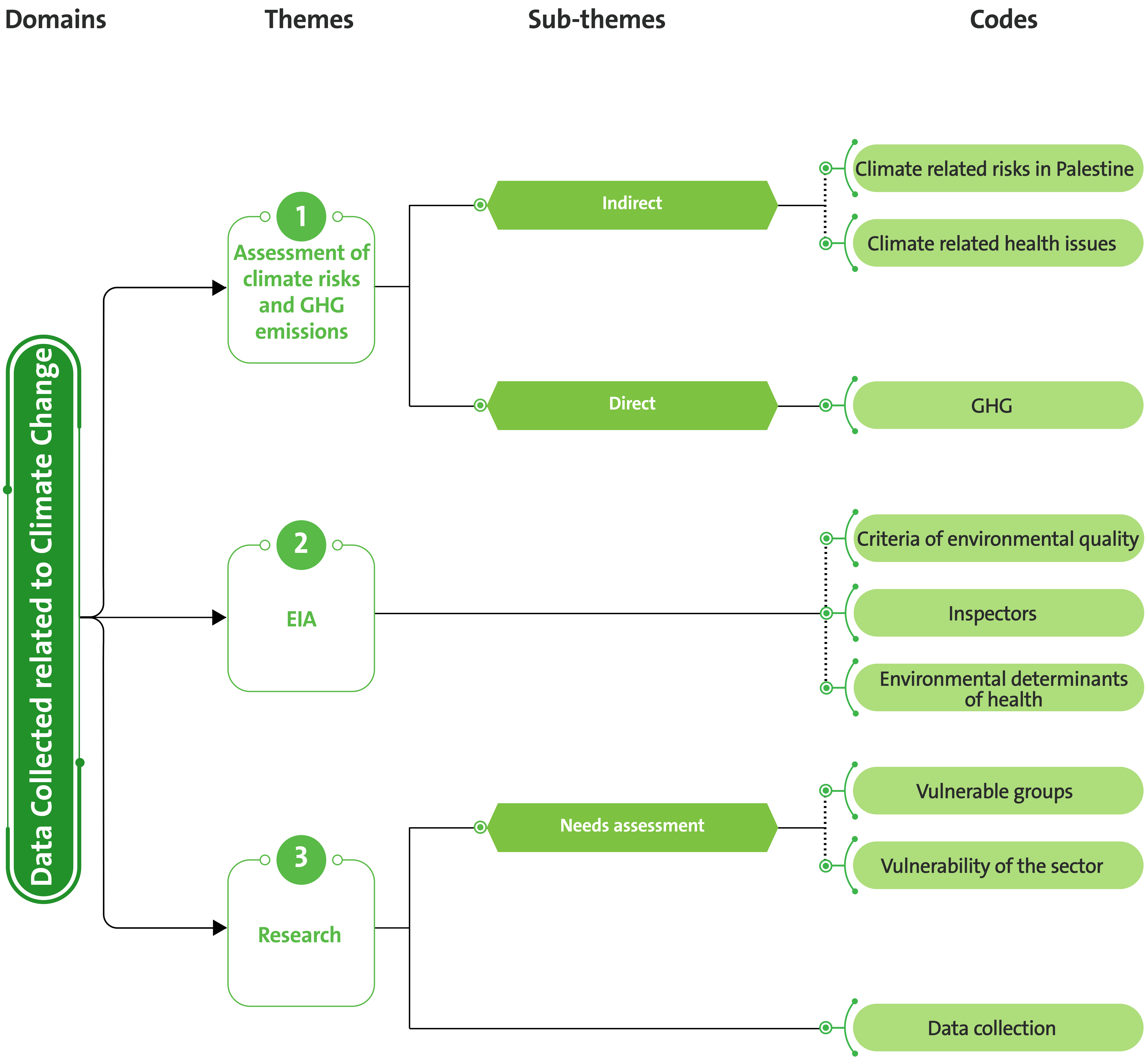

©2025 ICPH-BZU

The Current Situation of the Health System in Response to Climate Change in Palestine: Tree of Codes

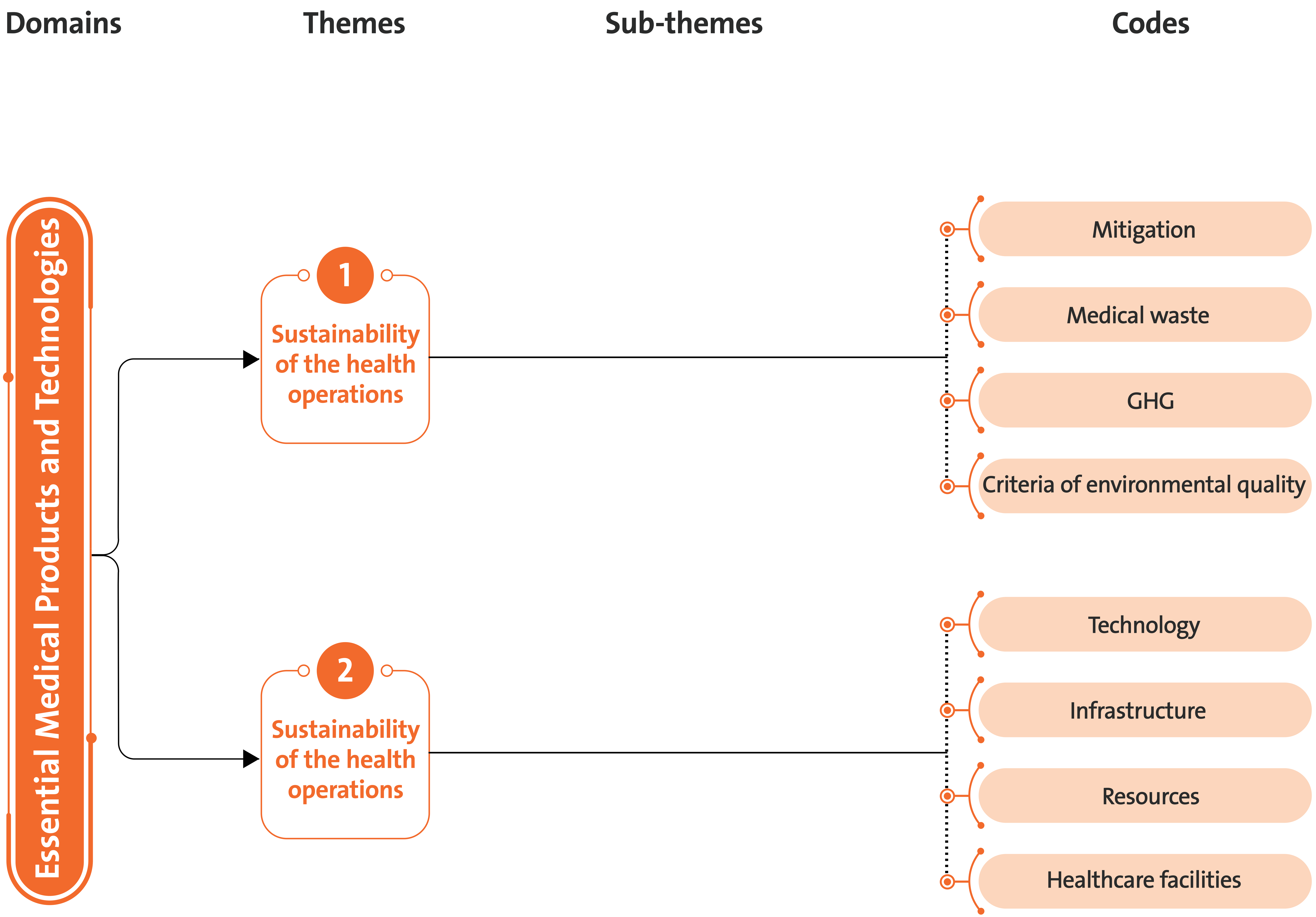

©2025 ICPH-BZU

The Current Situation of the Health System in Response to Climate Change in Palestine: Tree of Codes

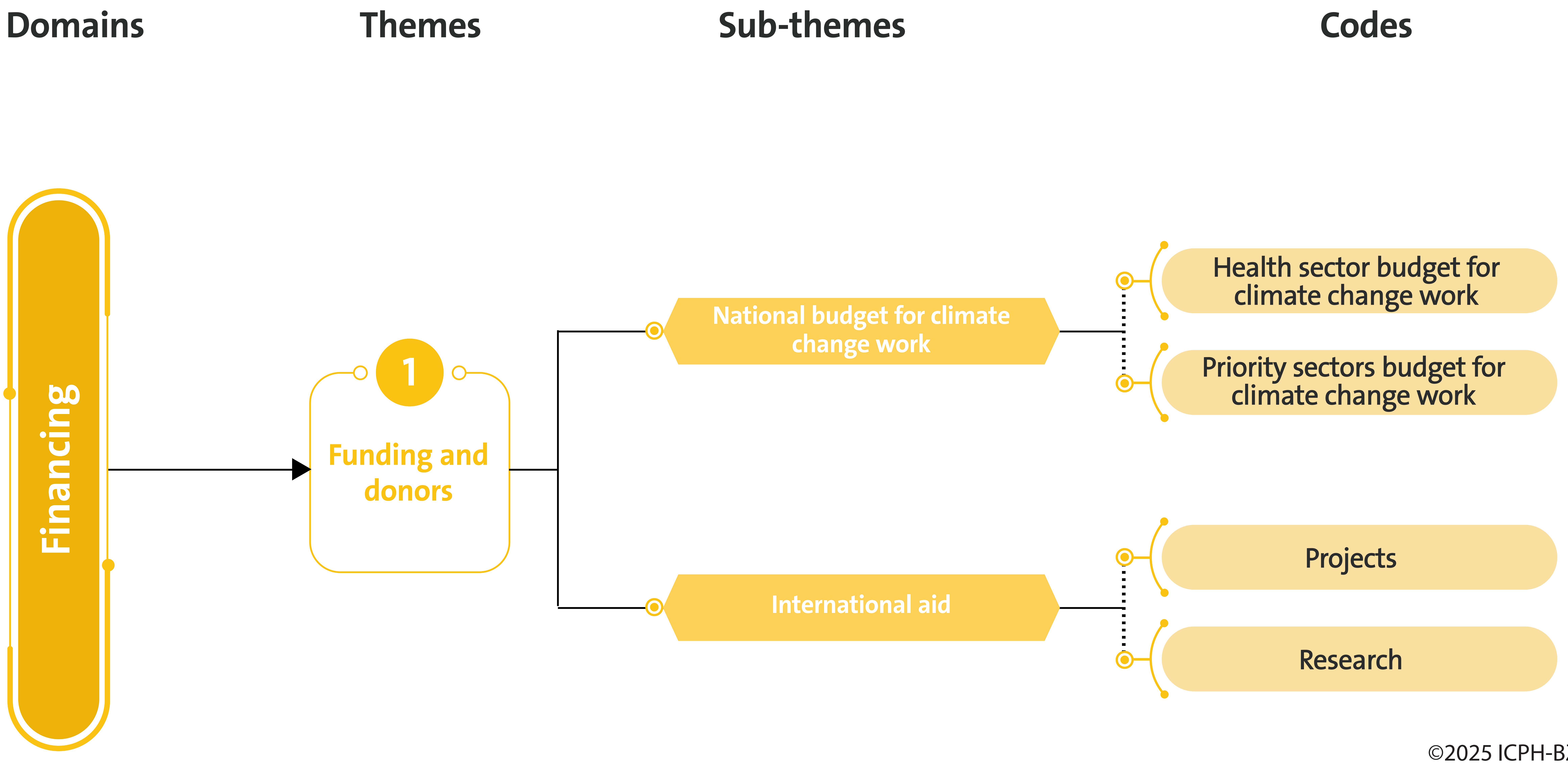

©2025 ICPH-BZU

The Current Situation of the Health System in Response to Climate Change in Palestine: Tree of Codes

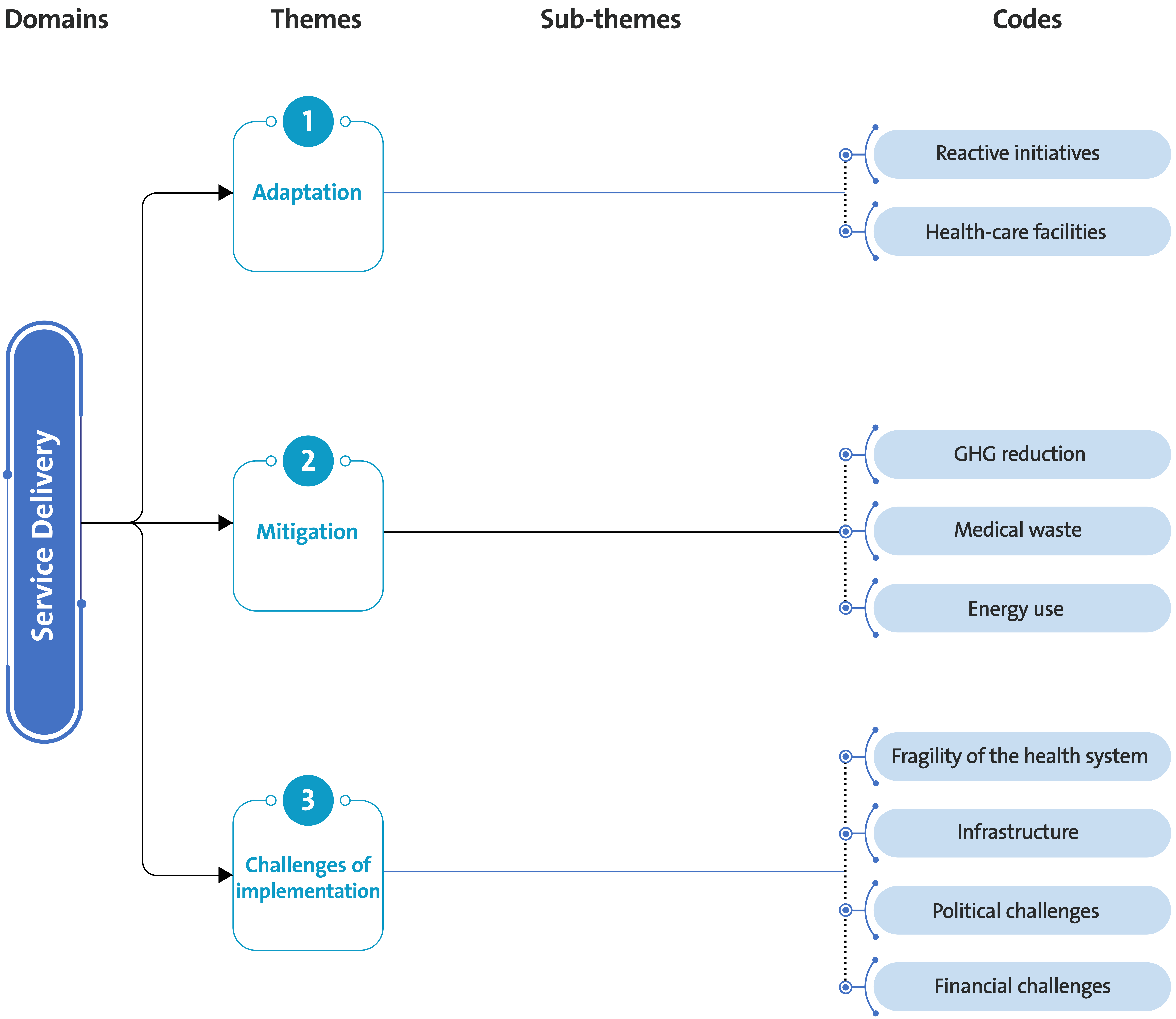

©2025 ICPH-BZU
